# The Potential of Bioaugmentation-Assisted Phytoremediation Derived Maize Biomass for the Production of Biomethane via Anaerobic Digestion

**DOI:** 10.3390/plants12203623

**Published:** 2023-10-20

**Authors:** Ana M. Paulo, Nídia S. Caetano, Ana P. G. C. Marques

**Affiliations:** 1 CBQF—Centro de Biotecnologia e Química Fina—Laboratório Associado, Escola Superior de Biotecnologia, Universidade Católica Portuguesa, 4169-005 Porto, Portugal; apaulo@ucp.pt; 2 LEPABE—Laboratory for Process Engineering, Environment, Biotechnology and Energy, Faculty of Engineering, University of Porto, Rua Dr. Roberto Frias, 4200-465 Porto, Portugal; nsc@isep.ipp.pt; 3 ALiCE—Associate Laboratory in Chemical Engineering, Faculty of Engineering, University of Porto, Rua Dr. Roberto Frias, 4200-465 Porto, Portugal; 4 CIETI/ISEP—Centro de Inovação em Engenharia e Tecnologia Industrial/Instituto Superior de Engenharia, Politécnico do Porto, Rua Dr. António Bernardino de Almeida 431, 4249-015 Porto, Portugal

**Keywords:** phytoremediation, heavy metals, maize, soil microbiota, biomass, energetic valorization, anaerobic digestion

## Abstract

Anthropogenic behaviors are causing the severe build-up of heavy metal (HM) pollutants in the environment, particularly in soils. Amongst a diversity of remediation technologies, phytoremediation is an environmentally friendly technology that, when coupling tolerant plants to selected rhizospheric microorganisms, can greatly stimulate HM decontamination of soils. Maize (*Zea mays*) is a plant with the reported capacity for HM exclusion from contaminated soil but also has energetic importance. In this study, *Zea mays* was coupled with *Rhizophagus irregularis*, an arbuscular mycorrhizal fungus (AMF), and *Cupriavidus* sp. strain 1C2, a plant growth-promoting rhizobacteria (PGPR), as a remediation approach to remove Cd and Zn from an industrial contaminated soil (1.2 mg Cd kg^−1^ and 599 mg Zn kg^−1^) and generate plant biomass, by contrast to the conservative development of the plant in an agricultural (with no metal pollution) soil. Biomass production and metal accumulation by *Z. mays* were monitored, and an increase in plant yield of ca. 9% was observed after development in the contaminated soil compared to the soil without metal contamination, while the plants removed ca. 0.77% and 0.13% of the Cd and Zn initially present in the soil. The resulting biomass (roots, stems, and cobs) was used for biogas generation in several biomethane (BMP) assays to evaluate the potential end purpose of the phytoremediation-resulting biomass. It was perceptible that the HMs existent in the industrial soil did not hinder the anaerobic biodegradation of the biomass, being registered biomethane production yields of ca. 183 and 178 mL of CH_4_ g^−1^ VS of the complete plant grown in non-contaminated and contaminated soils, respectively. The generation of biomethane from HM-polluted soils’ phytoremediation-derived maize biomass represents thus a promising possibility to be a counterpart to biogas production in an increasingly challenging status of renewable energy necessities.

## 1. Introduction

All over the globe, large areas are widely polluted due to anthropogenic activities such as industries, mining and smelting, urbanization, waste discharge, or bad agricultural practices [1]. Amongst the existing trace elements, heavy metals (HMs) are contaminants of great concern as they are not degradable and, unless removed or mobilized, they are persistent in the environment following their introduction [2], being toxic and bioaccumulable in the food chain [3]. Zinc and cadmium are frequently found in these contaminants [4]. While Zn is essential for plant growth and physiology (namely being a co-factor of some enzymes, taking part in nitrogen metabolism and participating in cell proliferation) [5], Cd is non-essential and promotes growth inhibition and decrease of photosynthetic activity [6]. Nevertheless, both are naturally present in the environment, but due to the above-mentioned anthropogenic activities, they can reach hazardous levels and represent an important environmental and health issue [7]. As a response to this problem, several physical and chemical clean-up techniques have been developed. Nevertheless, these approaches are usually high-priced and affect the soil properties and microbiome [8]. Phytoremediation, a biologically based technology that relies on the abilities of plants and their associated microbiota to remediate disturbed environmental matrices, is being seen as a more economically viable and environmentally friendly remediation solution [9]. An alternative way of decreasing the environmental risk posed by HM-contaminated soils can be the application of metal-tolerant plants to stabilize the soil surface, which reduces metal dispersion via erosion and leaching to the soil’s deeper layers—phytostabilization [10]. Nevertheless, the application of such a strategy takes a long remediation time to render soils to the legal thresholds [11] and poses a disposal problem associated with the produced biomass throughout this time span [12]. To respond to these shortcomings of phytoremediation, a solution has been proposed: the use of the obtained biomass for obtaining valuable products through energy production [13]. 

Bioenergy has contributed so far to 10% of the world’s primary energy, and it has the potential to reach up to 30 to 50% in the year 2050 [14]. Several energy generation methods can be regarded, but the anaerobic digestion (AD) of such biomass with methane-rich biogas production has been reported as presenting several advantages: increased cost-effectiveness, higher biomass reduction, biogas recovery potential, low energy consumption for the operation, reduced harmful emissions, potential of recovery of contaminants in the digestate and further application of the later as a fertilizer [15,16]. Nevertheless, the process should be pondered prudently, as HMs are known to influence anaerobic bacterial activities, probably due to their deleterious role on the enzymes of these microorganisms [17]. 

A crop adequate for this type of valorization is *Zea mays* L.: it is a fast-developing and high-yield crop, having HM tolerance and accumulation abilities [18]. With an average annual productivity of 60 tons ha^−1^ (fresh weight), which can be translated into a biogas potential of ca. 200 Nm^3^ [19], it can represent an important contribution to the necessary escalation in the production of energy from renewable sources. However, when growing plants in degraded soils, fertility may be lower, and there are factors to be considered, such as metal tolerance and accumulation, as well as plant development and biomass production yields. Specific microbiota that can resist the toxicity of HMs can be associated with plants via soil inoculation to diminish the deleterious effects of the soil contamination on these factors—bioaugmentation-assisted phytoremediation [20]—namely plant growth promoting rhizobacteria (PGPR) and arbuscular mycorrhizal fungi (AMF), whose synergistic effect has shown to enhance plant establishment and development in contaminated environments [21,22]. When inoculating *Z. mays* with *Cupriavidus* sp. strain 1C2, a PGPR, *Z. mays* was capable of thriving and growing in soils contaminated with levels of up to 30 mg Cd kg^−1^ and 1000 mg Zn kg^−1^ [23,24]. Additionally, preceding studies have demonstrated that *Cupriavidus* sp. strain 1C2 possessed PGP attributes in vitro [25] and that it showed tolerance to Zn and Cd in liquid culture containing up to 500 mg L^−1^ [26]. The inoculation of *Z. mays* with *Rhizophagus irregularis* (AMF) has also been our former subject of study [21,27]. The prospect of stimulating plant development and tolerance to diverse abiotic stressors by applying combined inocula, congregating the benefits of AMF and PGPR, was also validated by previous findings and proven to be of significant usefulness, as symbiotic relationships can be shaped between the inoculated AMF and PGPR, as well as with the broader soil microbiome and with the plant itself [21,27].

Thus, the aims of this work were, first, to assess the differences in chemical and biological soil composition and on the establishment, growth yield, and HM tissue accumulation capacities of *Z. mays* in an industrialized soil polluted with Cd and Zn, under assisted growth conditions (addition of selected microbial inocula) by comparing with the development of *Z. mays* in a non-polluted soil used for agricultural purposes; second, to assess the potential of the produced biomass for energetic valorization via AD as a viable solution, assessing the biogas and biomethane production yields, obtained from the AD of plants grown in both tested soils in order to understand the effects of HM presence in the AD process. The study presents, therefore, a strategy of using a metal-contaminated, otherwise barren and impaired soil to successfully produce a high-value crop while promoting its remediation and further apply the generated biomass for energetic purposes via AD—which can represent a contribution to a solution of the question of food vs. fuel created by the biomass needs imposed to supply bioenergy production.

## 2. Results

### 2.1. Maize Biomass Production Yields

*Zea mays* plants grown in soil A presented higher biomass yields for roots and cobs compared to their growth in soil I. However, the same was not verified for stems (Table 1). Biomass production was superior for stems, followed by cobs and roots, independently from soil conditions. Therefore, maize plants were composed of ca. 82% stems, 11% cobs, and 7% roots after growth in soil A and composed of 93% stems, 4% cobs, and 3% roots when grown in soil I. The root biomass was ca. 53% lower when grown in soil I compared to soil A. The soil treatment also had an effect on cob formation. Cob biomass was ca. 59% lower in soil I when compared to soil A. Despite root and cob biomass reduction, overall maize biomass reduction was not observed when grown in soil I due to the high percentage of biomass retrieved from the stems. In the end, a higher percentage in the total plant yield (ca. 9%) was observed after growth in soil I in comparison to the one in soil A.

### 2.2. Metal Accumulation in Different Plant Sections

The levels of Cd and Zn present in the different plant tissues were measured (Table 2), and results show that Cd and Zn concentrations were significantly different (*p* < 0.05) between soil conditions, being higher in plants grown in soil I. For both HMs, the maximum accumulation was observed in roots, followed by stems and cobs, although cobs from plants grown in soil A did not contain Cd. There was a significant difference (*p* < 0.05) in both HM accumulations in plant sections obtained after growth in soil I. For *Z. mays* grown in soil A, the accumulation of Zn in roots and stems was not significantly different (*p* < 0.05), while Cd accumulation was significantly different (*p* < 0.05) between these two plant sections.

### 2.3. Zn and Cd Mobilization in Soils

Neither of the two HMs was detected in water extracts. On the other hand, Zn and Cd were quantified in the soil in NH4-Ac solutions at the start and at harvest (the finale of the trial) (Table 3). Cadmium seems to not be bioavailable in any of the samples, as it was not detected. The level of extractable Zn was significantly (*p* < 0.05) higher for soil I when compared to soil A. The values of extractable Zn suffered a significant (*p* < 0.05) increase at the finale of the experiment for both soil conditions (A and I).

### 2.4. Biomass Composition Analysis

**Figure 1:**
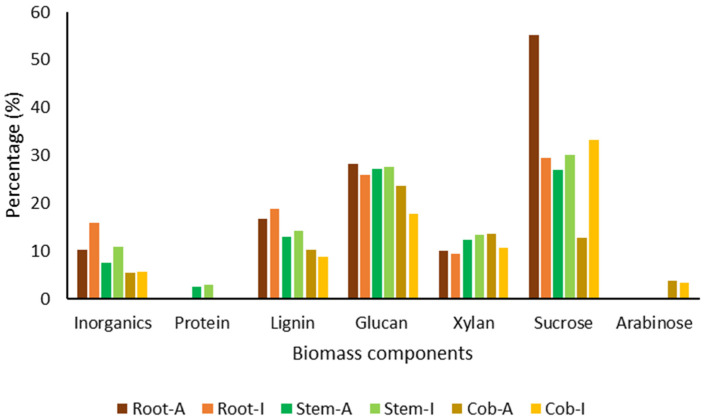
Biomass composition of the different plant parts.

As shown in Figure 1, biomass analysis allowed the identification of some differences in the composition of the different plant parts. The maximum percentage of inorganic compounds was identified in the roots, followed by the stems and cobs. The roots and stems obtained from plants grown in soil I contained a higher percentage of inorganic compounds. The protein content was quantified with a percentage between 2.5 and 2.9% in the stems, being detected in the other plant parts in percentages lower than 0.05%. Lignin was present in higher amounts in the roots, followed by the stems and the roots. Glucan was quantified in similar amounts between roots and the stems, being present in lower amounts in the cobs, mostly after growth in soil I. Xylan content was higher in the stems and cobs compared to the roots. Except for the very different values quantified in the roots and cobs, after growth in soil A, sucrose content was not significantly different between the different plant parts. Arabinose was only quantified in the cobs.

### 2.5. Microbial Community Changes in Soil

**Figure 2:**
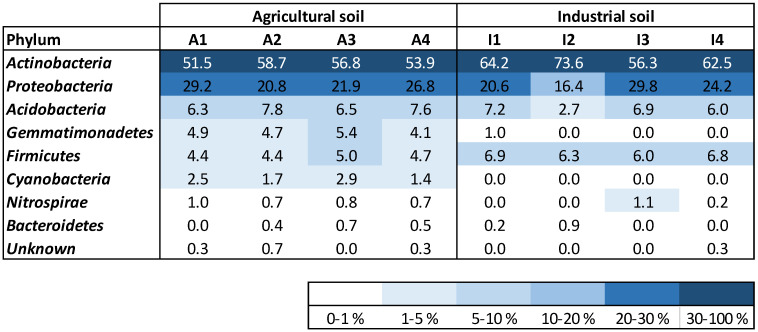
Density map of most abundant phylum of bacteria existent in the soil samples.

The bacterial community variety at the level of the phylum was discovered to be analogous in the two soil treatments (A and I), mostly in what concerns the profusion of Acidobacteria, Proteobacteria, and Actinobacteria (Figure 2). Actinobacteria governed the microbial community in the two soils, accounting for more than 52% and 56% of the total relative abundance in soils A and I, correspondingly. Proteobacteria represented between 16 and 30% of relative abundance in the two soil treatments. Acidobacteria were detected with a comparable relative richness in both soil conditions during Z. mays development (between 6 and 8%). Firmicutes were present in soil I with slightly higher relative abundance (ca. 6.5%) compared to soil A (ca. 4.5%). Gemmatimonadetes were not detected in most of the soil I samples but were present in all soil A samples, keeping a constant abundance until the end of plant development. Cyanobacteria were only identified in soil A (relative abundance between 1.4 and 2.9%). Nitrospirae were accounted for in higher amounts in soil A (ca. 0.8%) compared to soil I, with no bacteria from this phylum being detected at the start of the assay. Bacteroidetes were detected in soils A and I, with relative abundances between 0 and 0.7%.

**Figure 3:**
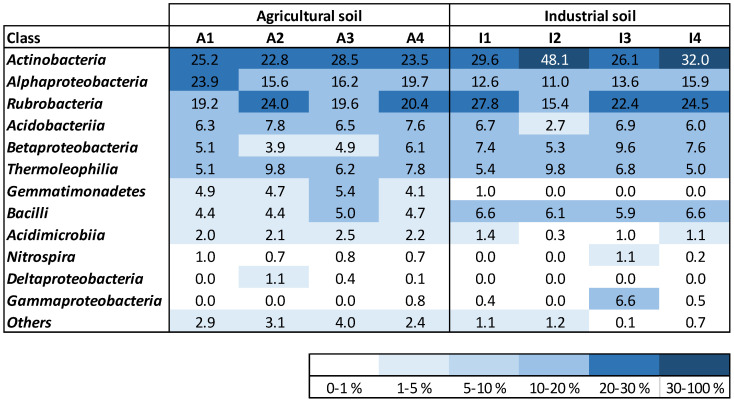
Density map of the most abundant bacterial classes existent in the soil samples; unknown and classes with relative abundance lower than 1% were gathered within the category “Others”.

Both soil treatments showed a high relative abundance of Rubrobacteria, Actinobacteria, and Alphaproteobacteria classes (Figure 3). Thermoleophilia and Acidobacteria accounted for a similar relative abundance at the start of maize growth and at harvest for both soil treatments. Betaproteobacteria were present in both soils (A and I) with similar relative abundance at the start of the experiment, increasing only after maize growth in soil I. Gemmatimonadetes were only identified in soil A, with no changes in the relative abundance throughout time. Bacilli were identified in the two soil treatments with slightly higher amounts in soil I. No important changes were observed in this bacterial class for soils A and I. Acidimicrobia were detected with higher richness in soil A. Nitrospira were present in soil A from the start to the end of the plant development, with similar relative abundance. This bacterial class was only detected in soil I at the end of the experiment. Deltaproteobacteria were only detected in soil A, while Gammaproteobacteria were mostly identified in soil I samples, although with low relative abundance.

**Figure 4:**
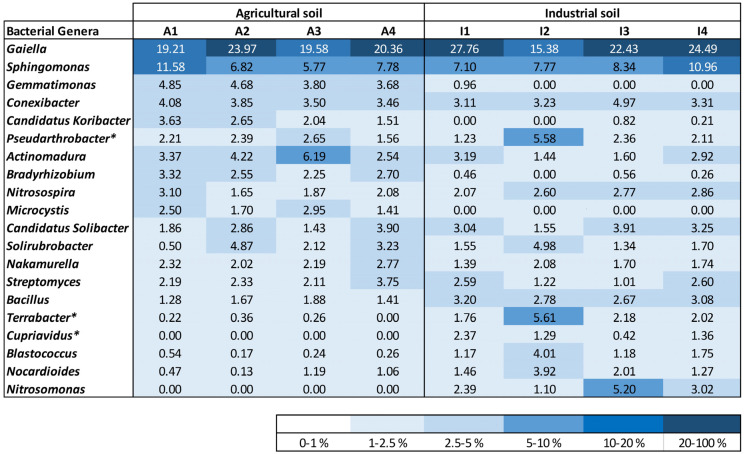
Density map with the most profuse genera of bacteria identified for the soil samples, taking into account the topmost 10 bacterial genera recognized in each soil sample. Bacterial genera designated with an asterisk (*) were additionally classified using BLAST from NCBI.

Governing bacterial genera were responsible for more than 60% of the total relative abundance in all analyzed cases. Some bacterial genera were present with similar relative abundance in both soil treatments (A and I) at the start and at the end of plant development, such as *Gaiella*, which was the dominant bacterial genus, *Sphingomonas*, *Conexibacter*, and *Nakamurella* (Figure 4). Other bacteria were mostly identified in one of the soil conditions. *Gemmatimonas*, *Candidatus Koribacter*, and *Microcystis* were mostly identified in soil A. On the other hand, *Terrabacter*, *Blastococcus*, and *Nitrosomonas* were only identified or found to be present with relative abundance higher than 0.5% in soil I samples. Bacteria belonging to the *Cupriavidus* bacterial genus were only identified in soil I, exhibiting a lower relative abundance in one of the duplicate samples collected at the end of the experiment. A higher amount of *Actinomadura* and *Bradyrhizobium* was identified in the agricultural soil, while *Nitrosospira*, *Bacillus*, and *Nocardioides* were acknowledged with superior relative abundance in soil I.

Some bacterial genera were found to change their relative abundance during the growth experiment. *Candidatus Koribacter* decreased while the relative abundance of *Nitrosomonas* increased at the end of plant growth in the A and I soils, respectively.

### 2.6. Root Colonization by AMF

Root colonization by AMF was registered for plants grown in both soil conditions. At harvest, plants grown in soil I (inoculated with both *Cupriavidus* strain 1C2 and *Rhizophagus irregularis*) showed a higher percentage of root colonization (49 ± 4) when compared to plants that developed in the non-inoculated soil A (29 ± 1). The significance (*p* < 0.05) of the difference between these mean percentages of colonization was confirmed with a *t*-test (t = 3.571).

### 2.7. Biogas Production

**Figure 5:**
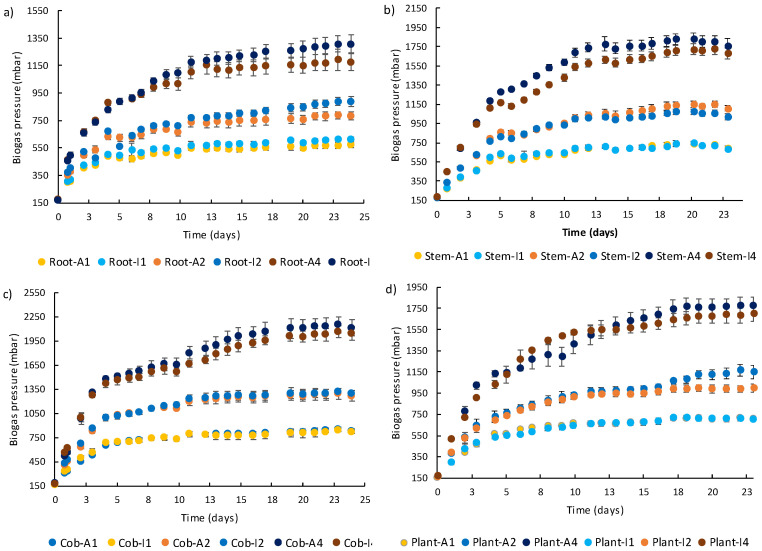
Cumulative biogas production obtained during anaerobic digestion of *Z. mays* roots (**a**), stems (**b**), cobs (**c**), and complete plants (**d**) after growth in agricultural (A) and industrial soil (I) at different inoculum-to-substrate (IS) ratios. Legend: 1—IS ratio 1:1; 2—IS ratio 1:2; and 4—IS ratio 1:4.

A higher amount of biogas and, consequently, methane was produced from a higher amount of plant tissue or complete plant for all IS ratios and both soil conditions (Figure 5), which resulted in significantly different results (Table 4, Table 5 and Table 6). Biogas production reached stable values in less than 3 weeks for IS ratios 1 and 2, requiring more time for IS ratio 4.

Table 4, Table 5, Table 6, Table 7, Table 8, Table 9 and Table 10 present comparative results obtained for several parameters (e.g., biogas pressure, volume, yield and IPR (initial biogas production rate), methane volume, and methane percentage in the biogas) measured at the end and start of the BMP assays, for all tested conditions. Statistical analysis (ANOVA) was executed for each soil treatment comparing plant sections and IS ratios; *t*-tests were performed between soil conditions for the same IS ratio and plant tissue (*p* < 0.001). Biogas production was significantly different between plant parts, being higher for the cob, followed by the stem and the roots (Table 4 and Table 5). Biogas production was not significantly different between the complete plant and stem alone for both soil conditions, excluding the biogas production obtained using the assay performed with IS2 from soil I. This is expected due to the higher contribution of stem biomass to the overall plant biomass. According to the *t*-test, no significant difference (*p* < 0.001) was found between biogas production for all IS ratios for roots, stems, cobs, and plants when comparing both soil conditions.

Biogas IPR was found to increase with the IS ratio within each soil condition, with significant differences for most of the tested conditions (Table 6). This increase was greater between IS ratios 2 and 4, mostly for cobs, followed by stems and roots. The biogas IPR values obtained for the plant for soil A, and most of the values obtained for soil I, were close to the values obtained for the stem, with no significant difference. When comparing all biogas IPR values for both soil conditions, all plant parts, and IS ratios, the *t*-test results showed no significant difference (*p* < 0.001).

Methane production increased with the amount of substrate and followed a similar trend compared to biogas production, with a greater amount of methane being produced from the cobs, followed by the stems and the roots (Table 7). Despite observing that methane obtained at the end of the process was not always significantly different between cobs and stems, methane obtained from the plant was often found to be closer to the results from the stem for most of the tested conditions. According to the *t*-test, no significant difference (*p* < 0.001) was found between methane production for all IS ratios for roots, stems, cobs, and plants when comparing both soil conditions.

The methane percentage in the biogas was found to be significantly different between several conditions (Table 8), indicating a higher variability of methane composition in the final biogas. The greatest similarity found between methane composition in the produced biogas was obtained for soil A, all IS ratios of root and stem. Despite this, *t*-test results did not show a significant difference (*p* < 0.001) between methane composition when comparing all IS ratios for roots, stems, cobs, and plants for both soil conditions, except for cob and IS ratio 2, where a significant difference was found (*p* < 0.001).

The biogas yield was higher for cobs, followed by stems and roots, as these values were found to be significantly different (Table 9). The complete plant presented a biogas yield with no significant difference compared to the values obtained for the stem, excluding results obtained for soil I, IS ratio 2 (Table 9). Despite some dissimilarities, the *t*-test results showed that no significant difference (*p* < 0.001) was observed for the obtained IS ratios for biogas yield obtained from roots, stems, cobs, or total plants when comparing both soil conditions.

Excluding some exceptions, the methane yield was higher for cobs, followed by stems and roots, presenting significantly different values (Table 10). Some methane yield values were significantly different from each other for different IS ratios and the same plant part. When comparing all methane yield values obtained for both soil conditions, different IS ratios, and plant parts, the *t*-test results showed no significant difference (*p* < 0.001).

## 3. Materials and Methods

### 3.1. Soil, Maize Development and Experimental Settings

Two types of soil were collected for this study from the North of Portugal with different HM contamination (Table 11): a soil used for agricultural purposes and an industrialized soil from Estarreja. The industrial soil has been contaminated for several years with HMs from discharges of solid residues and industrial wastewater [26]. Soil was collected randomly in the selected area to a 40 cm depth. The agricultural soil was used as a control soil since it contains less than 1 mg Cd and 300 mg Zn per kg of dry soil, according to the definition of non-contaminated soils by Kabata-Pendias [28]. Therefore, the results obtained during the phytoremediation strategy applied to the industrial soil were compared against the ones obtained for the agricultural soil, providing optimal conditions for maize development.

Two different conditions were tested: agricultural soil (A) and industrial soil inoculated with plant growth-promoting microbiota (I). For both conditions, 1 ton of each type of soil was placed in 1 m^3^ pots (1 m × 1 m × 1 m). As a bioaugmentation strategy, *Rhizophagus irregularis*—an arbuscular mycorrhizal fungi (AMF) used in other studies [21,27,30]—and *Cupriavidus* sp. strain 1C2 to be used as PGPR [31] were inoculated in the industrial soil. A volume of 10 L of commercial AMF inoculum (INOQ, GmbH, Schnega, Germany) was supplemented to the industrial soil before planting and mixed within the 20 cm of topsoil throughout the 1 m^2^ surface of the pot, while 10 L of sterile vermiculite was integrated into the agricultural soil in a similar way. The bacterial strain 1C2 was grown at 150 rpm and 30 °C in Luria–Bertani’s (LB) medium overnight. Bacterial pellets were then rinsed twice and re-suspended in 10 mM phosphate buffer pH 8.0 to obtain a concentration of ca. 108 CFU ml^−1^. After seedling emergence, 2 L of the bacterial solution was used to inoculate the industrial soil, evenly distributed throughout the 1 m^2^ of the surface of the soil, and 2 L of sterilized phosphate buffer was added to the agricultural soil.

Maize seeds (LusoSem, Algés, Portugal) were surface disinfected with 0.5% (*v*/*v*) NaOCl for 10 min and were afterward rinsed with sterilized deionized water. All seeds were propagated directly in the tested soils and, after germination, were reduced to 100 per pot (at a distance of 10 × 10 cm).

### 3.2. Plants Biomass Yield and HM Accumulation

Maize cuttings were collected 120 days after seedling emergence, divided into cobs, stems (consisting of shoots and leaves), and roots, and cleansed using diH_2_O and subsequently with HCl 0.1 M. All plant sections were submitted to air and successively to oven drying for 48 h at 70 °C to obtain the dry weight of the grown biomass per 1 m^3^ (1 ton) pot. Afterward, dry plant biomass was ground and digested according to USEPA methodology 3052 using a PerkinElmer MicroWave 3000. Zinc and Cd levels of the digests were assessed in a Unicam 960 spectrophotometer (Waltham, MA, USA) by Flame Atomization-Atomic Absorbance Spectrometry (FA-AAS) of the digests [32], with detection limits of 0.0033 mg L^−1^ for Zn and 0.0028 mg L^−1^ for Cd. A reference sample (CRM 279, sea lettuce) was selected according to the Community Bureau of Reference (CBR) and was evaluated by applying the aforementioned Zn and Cd assessment methodology. The results registered by FA-AAS (52.8 ± 0.9 and 0.28 ± 0.01 mg kg^−1^ for Zn and Cd correspondingly) established the accurateness and exactitude of the methodology when compared with the validated value (51.3 ± 1.2 mg and 0.274 ± 0.022 kg^−1^ for Zn and Cd correspondingly).

### 3.3. Heavy Metal Mobilization in Soil

Soil was collected at a depth of 10 cm from each pot (A and I) using a soil sampler at start and at harvest to evaluate the ammonium acetate (NH_4_-Ac) and the water-extractible Zn and Cd portions. Mixtures of 1:5 soil water and 1:5 soil NH_4_-Ac [33] were maintained at 20 °C with rotation for 2 h. The extracts were then centrifuged at 38,000 rpm for 10 min, and supernatants were clarified over a 0.45 µm cellulose acetate filter. Zinc and Cd levels were assessed using the FA-AAS, as previously described.

### 3.4. Biomass Composition

The contents of ash, proteins, lignin, xylan, sucrose, arabinose, and cellulosic glucan existent in *Z. mays* roots, stems, and cobs were measured according to standard methods described by NREL (https://www.nrel.gov/), by using the available online protocols: determination of the content of ash (https://www.nrel.gov/docs/gen/fy08/42622.pdf), protein (https://www.nrel.gov/docs/gen/fy08/42625.pdf), structural carbohydrates and lignin (https://www.nrel.gov/docs/gen/fy13/42618.pdf), sugar byproducts (https://www.nrel.gov/docs/gen/fy08/42623.pdf) and cellulosic glucan (https://www.nrel.gov/docs/fy21osti/76724.pdf) in biomass (accessed on 20 February 2022).

### 3.5. Soils Bacterial Community Analysis

DNeasy PowerSoil Kit (Qiagen, Hilden, Germany) was used according to manufacturer’s instructions for extracting DNA from duplicate soil samples, collected at the start and at the end of plant development for treatments A and I (1 and 2 are replicate soil samples collected at the start of plant development; 3 and 4 are replicate soil samples collected at harvest). The DNA concentration was assessed using Qubit (Thermo Fisher Scientific, Waltham, MA, USA), and the obtained DNA was saved for further utilization at −20 °C.

The obtained DNA was used for performing Next Generation Sequencing (NGS) and bioinformatics data analysis (GATC-Eurofins, Konstanz, Germany). The sequencing of the 16S rRNA gene was completed to comprise the V3–V4 hypervariable zone (Illumina MiSeq program) by making use of two primers (357F—TACGGGAGGCAGCAG [34] and 800R—CCAGGGTATCTAATCC) [35]. The bacterial community analysis and description were performed as explained by Paulo et al. [36]. The operational taxonomic unit (OTU) sequences of bacterial families presenting high relative abundance, but which could not be classified further at the level of the genus, were subjected to a comprehensive identification using BLAST from NCBI (https://blast.ncbi.nlm.nih.gov/Blast.cgi (accessed on 22 March 2022)).

### 3.6. AMF Colonization of Roots

A portion of newly harvested thin roots was gathered from the maize plants collected from both soil treatments (A and I) at harvest. Roots were cut into approximately 1 to 2 cm sections, submerged in KOH 10% (m/V), and warmed for 30 min in water bath at 80 °C, complying with a method adjusted from the study of Vierheilig et al. [37]. Afterward, the remainder of the solution of KOH was discharged, and roots’ pieces were cleared by adding HCl 3% (*v*/*v*) and letting it rest for 10 min. Subsequently, the fragments were colored using a staining solution consisting of 5% ink (Pelican 4001, Brilliant black, Fountain Pen Ink) diluted in 5% (*v*/*v*) CH₃COOH and simmered at 80 °C for 4 min, followed by washing the roots repeatedly with water. Tainted roots were observed under the microscope to calculate the percentual fraction of AMF colonization by applying the grid-line intersect methodology [38].

### 3.7. Anaerobic Digestion of Zea mays Biomass

Dried and ground roots, stems and cobs, and the complete plant from both soil conditions (A and I) were used as substrates for biomethane (BMP) assays. Serum bottles (120 mL) were filled with anaerobic medium (45 mL), and the headspace was flushed with 80% N_2_: 20% CO_2_ mixture used for the anaerobic digestion assays, in agreement with Angelidaki et al. [39]. The anaerobic granular sludge used in all the assays (1 g wet weight; 0.089 g VS) was collected from a full-scale EGSB (Expanded Granular Sludge Bed) reactor used for wastewater treatment at a beverage company located at Matosinhos (Portugal). Sodium acetate, 20 mM (Sigma-Aldrich, St. Louis, MO, USA), was used as substrate for obtaining biomass-specific methane activity. Daily methane production was measured until stabilization to determine the anaerobic granular biomass acetoclastic activity. In order to test increased substrate addition and its effect on anaerobic digestion, different inoculum-to-substrate ratios (VS-based) were evaluated in triplicate, namely 1:1, 1:2, and 1:4 (indicated in the text as I:S 1, 2, and 4). A blank control, without substrate, was also tested in triplicate. Bottles were executed in triplicate, and bottles were incubated at 37 °C and agitated once a day. A pressure meter (Paralab, Valbom, Portugal) was used for measuring daily biogas production until stable values were obtained. Biogas production (volume per batch assay) was calculated from pressure values after subtracting biogas produced in blank assays. The composition of biogas in terms of methane was determined using a gas chromatograph, Varian CP-3800, with a TCD detector (Agilent Technologies, Santa Clara, CA, USA) and a Carboxen^®^-1006 PLOT column (30 m × 0.53 mm I.D.; Merck, Darmstadt, Germany). Hydrogen gas was used as carrier gas, and a standard gas mixture composed of methane, nitrogen, and carbon dioxide (40:40:20%*v*/*v*) was used to calculate the different biogas components. Total solids (TS) and volatile solids (VS) of the anaerobic granular biomass and *Z. mays* plant parts were quantified using standard methods [40].

### 3.8. Statistical Analysis

Differences between the different parameters obtained for both conditions A and I were statistically analyzed by one-way ANOVA and *t*-tests using the IBM SPSS Statistics program (IBM, Armonk, NY, USA, version 28.0). Duncan test (*p* < 0.05) was executed to ascertain the significance of the differences among the means.

### 3.9. Chemical Reagents

The chemicals used were of analytical grade and were obtained from Pronalab (Sintra, Portugal) and Promega (Madison, WI, USA) for liquid reagents and Sigma Aldrich (St. Louis, MO, USA) and Merck (Darmstadt, Germany) for solid reagents.

## 4. Discussion

A phytoremediation strategy for promoting the growth of *Z. mays* in soil from an industrial region was performed, together with the energetic valorization of the complete plant and plant tissues through biogas production. Phytoremediation and biogas production results analysis have shown that plant growth conditions did not affect either plant biomass or biogas production yields. Plants growing in the industrial soil were exposed to levels of 599 mg Zn kg^−^^1^ and 1.2 mg Cd kg^−^^1^, which are close to or even above those considered as concerning in soils according to the Canadian Soil Quality Guidelines (200–360 mg Zn kg^−1^ and 1.4 mg Cd kg^−^^1^). Total *Z. mays* biomass production was not affected by the HMs present in soil I, with there being a decrease in root and cob biomass yields but not of the stem, which contributes to a greater *Z. mays* biomass proportion. Although the presence of Zn and Cd in the soil can impose disturbances in the plant’s metabolic and physiological mechanisms, which ultimately can result in plant yield reduction [5,41], this detrimental effect might be more relevant in the *Z. mays* roots, which are in closer contact with the contaminated soil. Nevertheless, Zn uptake by the plants grown in soil I was more distributed between the root (ca. 50%) and the aerial parts (ca. 50%), while Cd was accumulated to a higher extent in the maize roots (>66%), compared to the aerial part. This agrees with the higher content of inorganic compounds quantified in roots and stems from *Z. mays* grown in soil I, in comparison to plants grown in soil A. Higher HM accumulation in the roots than in the stems was previously reported for maize in other studies [21]. Also, similarly to the report of Meers et al. [19], Zn and Cd concentrations in the grains (cob) were the lowest in both soil scenarios. It has been suggested that this avoidance of metal translocation to the aboveground tissues may be a protective strategy against toxification of its reproductive organs and, consequently, to ensure its descendants [19,42].

The effect of Zn and Cd in maize yields was accompanied by an intensification in the tissue accumulation of both targeted HMs, reaching levels up to 448 mg Zn kg^−^^1^ and 10.3 mg Cd kg^−^^1^ in maize roots. The range of metal concentrations for *Z. mays* grown in the industrial soil described in the present report is in accordance with other studies in the literature. Guo et al. [43] reported grain accumulations of ca. 57 to 73 mg Zn kg^−^^1^ and 0.17 to 0.31 mg Cd kg^−^^1^ while showing shoot accumulations of 577 to 779 mg Zn kg^−^^1^ and 1.9 to 3.1 mg Cd kg^−^^1^ for a soil with 246 mg Zn kg^−^^1^ and 0.96 mg Cd kg^−^^1^; Adewole et al. [44] reported accumulations of up to 307, 231, and 58 mg Zn kg^−^^1^ for maize roots, shoots and grains, respectively, for a soil contaminated with ca. 788 mg Zn kg^−^^1^; Moreira et al. [21] showed that maize accumulated up to 478 and 239 mg Zn kg^−^^1^ in its roots and shoots, respectively, and Cd was not detectable when grown in a mining soil presenting accumulations of 286 mg Zn and 9.7 mg Cd per kg of plant dry weight. In our study, about 50% of the amount of Zn uptake by the plant was quantified in the root and the remaining in the stem and cobs, independently of soil condition, indicating that the industrial soil contamination did not interfere with the mechanisms of Zn uptake by maize. Although the effect of HM accumulation on the plant’s biomass composition and HM uptake was not clearly observed, a greater variation in sucrose levels was identified between *Z. mays* tissues and soil conditions, possibly because of metabolism adaptation strategies. Sugars can play a key part in plant defense mechanisms against various biotic and abiotic stress influences, with sucrose being essential for sugar exchanges in higher plants [45].

Additionally, the growth of maize in the tested soils induced an increase in the soluble fractions of Zn in particular—probably due to the production of organic acids in the rhizosphere of the growing plants [46], which ultimately acidify the soil and solubilize the present metals [47].

Despite these HM accumulation levels, *Z. mays* was able to thrive and generate more biomass when exposed to the stress imposed by the high contamination levels present in soil I. In fact, an increase in total plant yield of ca. 9% was observed after growth in the microbiota-amended industrial soil compared to the agricultural soil. Plants possess their own mechanisms to withstand exposure to HM contaminants by producing sequestering molecules [48], controlling their growth regulators [49], or improving their antioxidant systems [50]. However, based on our previous knowledge of the positive effects of the inoculation with the selected microbiota *R. irregularis* and *Cupriavidus* sp. strain 1C2 on the growth and metal resistance abilities of *Z. mays* [21,23,25,31], this application can also help explain the results obtained for the plants grown soil I. Plant growth-promoting rhizobacteria can stimulate plant growth by (1) providing phytoavailable nutrients to the plant host (through phosphorous solubilization, nitrogen fixation, and iron carrier production [51]; (2) protecting against pathogens [52,53]; (3) producing phytohormones and specific enzymes such as gibberellin (GA), indole-3-acetic acid (IAA), abscisic acid (ABA), ACC-deaminase, and ethylene (ETH), which mediate various physiological processes in plants [54]; (5) inducing systemic resistance by activating essential antioxidant enzymes, namely catalase (CAT), superoxide dismutase (SOD), and peroxidase (POD) [51]; and by (6) helping plants endure stress caused by metal exposure by accumulating, transforming and detoxifying metals in the rhizosphere [55] (converting toxic HM ions to less toxic ions by sequestering and absorbing extracellular polymers [56] and methylation and redox reactions [57]. This positive effect of bacterial inoculation was observed in maize plants grown in HM-contaminated soils in previous studies, namely in Cu-contaminated farmland [46], in Cd- and Zn-amended soils [24], and in mine land [21]. By following the bacterial community during the study, it was perceived that the inoculated bacteria *Cupriavidus* sp. strain 1C2 prevailed in the rhizosphere of *Z. mays* samples grown in the industrial soil, which might have contributed to the observed results. The higher AMF colonization rates observed for roots of maize plants grown in soil I indicate a good proliferation of the applied *R. irregularis* inocula on this soil and an active symbiosis between the fungi and the plant [58] that can also explain its positive biomass production results. A positive effect was observed when maize was inoculated with selected AMF species, namely when growing exposed to Pb [59], Cd, Cr, Ni [60], Cd [21,61], and Zn [21] contaminated soils. This happens as AMF can also improve plant growth and response to metal toxicity, either through nutritional benefits such as increased water absorption and nutrient uptake due to increased absorptive surface area of plant roots and solubilization and/or mineralization processes, physiological changes in the host plant such as the production of phytohormones (like cytokinins and gibberellins) and other metabolites (amino acids and vitamins), or the improvement of its antioxidant system (by affecting the expression of antioxidant enzymes) and osmoregulation (enhancing carbon dioxide exchange rate, water use efficiency, and stomatal conductance) [20,62,63], which will ultimately improve the plant’s protection against HM exposure.

*Zea mays* rhizosphere was analyzed to understand if the phytoremediation strategy affected the microbial community composition and if these changes could be used as bioindicators of soil quality at the end of the phytoremediation strategy. Overall, results indicate that plant–soil interaction did not affect the soil microbiome during *Z. mays* growth in soil A. This observation was also true for soil I, indicating that the phytoremediation strategy did not induce a major variation in the overall microbial community present in maize’s rhizosphere. However, the observation of a greater microbial variability at the bacterial genera level can be associated with different soil quality conditions between soils and experimental points. Several bacterial genera, known to be able to thrive in HM-contaminated soils, were found to be present in similar amounts in both soils, namely *Gaiella* [64], *Sphingomonas* [65], *Conexibacter* [66], *Pseudoarthrobacter* [67] and *Candidatus Solibacter* [68]. *Gaiella* and *Sphingomonas* were found in the rhizosphere and were associated with a higher resistance of strawberry cultivars to pathogenic fungi [69], having an important role in plant protection. *Pseudoarthrobacter* is a bacterium also present in plants’ rhizospheres and is considered a PGPR [67]. Some bacterial genera presented a different pattern between soil conditions. For example, *Gemmatimonadetes* is a bacterial genus present in the soil, associated with C/N cycling processes in soil, and known for its HM-tolerance [70], but was only identified in soil A. Similarly to *Candidatus Solibacter*, *Candidatus Koribacter* is associated with soil carbon and sulfur metabolism and was also identified in HM-contaminated soils [68]. But differently from *Candidatus Solibacter*, *Candidatus Koribacter* was present in soil A but was only detected in soil I at the end of the plant’s growth. Ammonium-oxidizing bacteria (AOB) and nitrite-oxidizing bacteria (NOB) are nitrifiers associated with the conversion of ionic nitrogen forms to atmospheric nitrogen in the soils. Since AOB and NOB can be quite sensitive bacterial groups, these can be applied as bioindicators of HM pollution [71]. *Nitrosospira* (AOB) was identified in both soil conditions, while *Nitrosomonas* (AOB) was only detected in soil I, increasing in relative abundance at the end of the phytoremediation strategy. *Nitrospira* (NOB) kept its relative abundance in soil A (between 0.7 and 1%) but increased from zero to values between 0.2 and 1% at the end of plant development in soil I. Concurring with these results, some bacteria might have been able to improve their numbers after the phytoremediation strategy application. *Nitrosomonas* and *Nitrospira* dynamics, for example, can be an indication of a more amenable environment in soil I generated by the phytoremediation strategy. More analysis regarding the microbial dynamics of HMs of industrial and non-contaminated soils is required in order to better understand how soil microbial dynamics can be related to soil quality.

When comparing the accumulation levels obtained for the aboveground tissues with the European standards for animal feed (European Commission Directive 2002/32/EC)—1.14 mg Cd kg^−^^1^ and no limit for Zn—the cobs from plants growing in soil I used in this study can even be used for this purpose. However, the installed agronomic practices generally do not encompass simultaneous harvesting and separation of grains—the options are generally harvesting the entire plant or only harvesting the cobs with the incorporation of the remainder tissues to the land [19]—which is not a desirable option when establishing a phytomanagement strategy, as we want to remove the contaminated biomass. Other utilizations should be designed for the recovered biomass, and anaerobic digestion is potentially an appropriate one as it is viable for all plant sections. According to our results, biogas production yields from *Z. mays* used for soil I phytoremediation were not affected by the uptake of HMs by the plants when compared to the control condition (growth in soil A). Increasing the amount of available substrate is intended to also increase the release of HMs resulting from plants’ biodegradation and check for possible process inhibition. The pH, HM concentration, chemical form, and redox potential [72,73], besides many other HM physical–chemical characteristics, can determine its behavior in a biological process, affecting its adsorption onto the biomass and effect on the anaerobic digestion [16,74]. Zinc is necessary for the catalysis of several anaerobic reactions occurring in methanogenic archaea species [75], and a concentration of up to 1250 mg Zn L^−^^1^ was discovered to intensify biogas generation through swine manure anaerobic digestion [73]. This occurred with a much higher Zn level compared to the maximum expected to be released from the anaerobic digestion of maize roots (ca. 4.1 mg Zn L^−^^1^, IS ratio 4). However, Zn toxicity in anaerobic digestion was also observed for much lower concentrations. For example, Guo et al. [76] referred to a different toxicity potential associated with Zn, present in a solution with a 3 mg Zn L^−^^1^ concentration. Cadmium, on the other hand, was also found to be toxic to the anaerobic digestion processes at different concentrations: 1 mg Cd L^−^^1^ and 36 mg Cd L^−^^1^ [72,76]. These concentrations are also higher compared to the ones predicted from the release of Cd during the anaerobic digestion of maize roots (ca. 0.1 mg Cd L^−^^1^, IS ratio 4). It was also found that Zn can have a greater effect on the anaerobic digestion when using suspended sludge, being toxic at 0.5 or 7.5 mg L^−^^1^ towards anaerobic suspended or anaerobic granular sludge, respectively [72,77]. In the anaerobic granular sludge, layers of bacterial biofilm shelter the most sensitive anaerobic microbes (e.g., methanogenic bacteria) [72]. The fact that granular sludge was used in this study might also explain the results obtained. The use of the complete plant or its aerial part for energetic valorization through biogas production can be a good energetic valorization route. The last option might be preferred since the aerial part of the maize (stems and cobs) constitutes between 92 and 97% of the total plant dry weight, and the current agricultural practices remove the plant but leave the roots in the soil. Besides the energetic valorization, using the anaerobic digestion process will also help reduce the volume of removed plants [16].

## 5. Conclusions

An optimized strategy for creating the best conditions for *Z. mays* growth in industrial soil contaminated with 1.2 mg Cd and 599 mg Zn per kg of plant dry weight was implemented, aiming at soil phytoremediation followed by maize biomass valorization via anaerobic digestion. The strategy consisted of growing the *Z. mays* plants in the contaminated soil with simultaneous inoculation with the arbuscular mycorrhizal fungus *Rhizophagus irregularis* and the plant growth-promoting rhizobacteria *Cupriavidus* sp. strain 1C2. As a benchmark, the same plant was grown in non-contaminated soil without any additional inoculation. It was concluded that about 50% of the amount of Zn uptake by the plant was accumulated in the root and the remaining in the stem and cobs, independently of soil. The biomass fractions corresponding to roots, stems, and cobs were significantly different for the plants grown in both soils, with 93% (82%) of stems, 3% (7%) of roots, and 4% (11%) of cobs for industrial and agricultural soil. Regarding the second objective of this research, it was concluded that neither biogas nor methane production for roots, stems, cobs, and plants was significantly different when comparing biomass from plants grown in both soils. Finally, the strategy of soil phytoremediation using *Z. mays* combined with *Rhizophagus irregularis* and *Cupriavidus* sp. strain 1C2, followed by biomass conversion to biogas (biomethane) through anaerobic digestion, shows great potential.

## Figures and Tables

**Table 1:** *Zea mays* biomass yields.

Treatment	Biomass (g per Pot)		
	Root	Stem	Cob
Agricultural (A)	127.51	1473.45	203.81
Industrial (I)	59.55	1822.12	86.17

**Table 2:** Metal accumulation for the Z. mays tissues.

Treatment	Zn (mg kg^−1^ Dry Weight)				Cd (mg kg^−1^ Dry Weight)			
	Root	Stem	Cob		Root	Stem	Cob	
Agricultural (A)	72 ± 5 ^A,a^	61 ± 8 ^A,a^	13 ± 3 ^A,b^	*** F_3,6_ = 98.055	1.4 ± 0.2 ^A,a^	0.6 ± 0.2 ^A,b^	n.d. ^A,c^	*** F_3,6_ = 39.283
Industrial (I)	448 ± 13 ^B,a^	421 ± 12 ^B,b^	66 ± 9 ^B,c^	*** F_3,6_ = 1,083,382	10.3 ± 0.6 ^B,a^	4.7 ± 0.6 ^B,b^	0.41 ± 0.01 ^B,c^	*** F_3,6_ = 648,310
	t = 4.597	t = 1.451	t = 5.618		t = 1.604	t = 9.583	t = 16.000	

Values are displayed as averages ± standard deviation (n = 3). For every soil treatment and for every HM, one-way ANOVA was executed, and test outcomes (F) are presented in related lines as ***—significant at *p* < 0.001 level. Averages for equal soil treatment exhibiting diverse lowercase letters are significantly (*p* < 0.05) different from one another, in accordance with the Duncan test. *t*-tests for independent samples were executed for every type of plant tissue to assess the influence of soil treatment, and outcomes (t) are indicated in related columns. Averages in the same column presenting diverse uppercase letters are significantly (*p* < 0.05) different from one another.

**Table 3:** Zn and Cd in soil NH_4_-Ac extracts from soils collected at the start and at harvest.

Treatment	Zn (mg kg^−1^)			Cd (mg kg^−1^)	
	Start	Harvest		Start	Harvest
Agricultural (A)	18.8 ± 0.7 ^a,A^	27.2 ± 0.7 ^a,B^	t = 0.011	n.d.	n.d.
Industrial (I)	69 ± 2 ^b,A^	95 ± 2 ^b,B^	t = 0.680	n.d.	n.d.
	t = 0.837	t = 5.838		--	--

Values are displayed as average ± standard deviation (n = 3). *t*-tests for independent samples were executed for every HM to assess the influence of soil treatment, and outcomes (t) are indicated in related columns. Averages in the same column presenting diverse lowercase letters are significantly (*p* < 0.05) different from one another. *t*-test was also executed for every soil treatment, and outcomes (t) are indicated in related lines. Averages in the same line presenting diverse uppercase letters are significantly (*p* < 0.05) different from one another.

**Table 4:** Biogas pressure (mbar) obtained at the end of BMP assays (average ± SD).

Treatment	IS Ratio	Root	Stem	Cob	Plant	F_4,8_
**Agricultural (A)**	1	207 ± 14 ^a,A^	343 ± 20 ^b,A^	466 ± 20 ^c,A^	320 ± 7 ^b,A^	133.665 ***
	2	422 ± 23 ^a,B^	723 ± 31 ^b,B^	871 ± 52 ^c,B^	772 ± 65 ^b,B^	53.258 ***
	4	824 ± 54 ^a,C^	1424 ± 36 ^b,C^	1776 ± 85 ^c,C^	1435 ± 26 ^b,C^	155.465 ***
	F_3,6_	245.064 ***	1042.181 ***	390.523 ***	568.955 ***	
**Industrial (I)**	1	240 ± 14 ^a,A^	342 ± 5 ^b,A^	474 ± 8 ^c,A^	326 ± 25 ^b,A^	123.989 ***
	2	511 ± 40 ^a,B^	674 ± 5 ^b,B^	943 ± 41 ^c,B^	602 ± 2 ^d,B^	124.295 ***
	4	935 ± 65 ^a,C^	1326 ± 72 ^b,C^	1670 ± 80 ^c,C^	1343 ± 66 ^b,C^	53.616 ***
	F_3,6_	181.023 ***	432.873 ***	397.225 ***	504.720 ***	

Values are displayed as averages ± standard deviation (n = 3). For every soil treatment and for every IS ratio, one-way ANOVA was executed, and test outcomes (F) are presented in the related lines as ***—significant at *p* < 0.001 level. Averages for equal soil treatment and IS ratio exhibiting diverse lowercase letters are significantly (*p* < 0.05) different from one another in accordance with the Duncan test. For every soil treatment and every plant tissue, one-way ANOVA was also executed, and test outcomes (F) are presented in related columns and as ***—significant at *p* < 0.001 level. Averages for the same soil treatment and plant tissue presenting diverse uppercase letters are significantly (*p* < 0.05) different from one another in accordance with the Duncan test.

**Table 5:** Biogas volume (mL/batch) obtained at the end of BMP assays (average ± SD).

Treatment	IS Ratio	Root	Stem	Cob	Plant	F_4,8_
**Agricultural (A)**	1	13 ±1 ^a,A^	25 ± 1 ^b,A^	34 ± 1 ^c,A^	23.4 ± 0.5 ^b,A^	133.665 ***
	2	31 ±2 ^a,B^	53 ± 2 ^b,B^	64 ± 4 ^c,B^	56 ± 5 ^b,B^	53.258 ***
	4	60 ± 4 ^a,C^	104 ± 3 ^b,C^	130 ± 6 ^c,C^	105 ± 2 ^b,C^	155.465 ***
	F_3,6_	245.064 ***	1042.181 ***	390.523 ***	568.955 ***	
**Industrial (I)**	1	18 ± 1 ^a,A^	25 ± 0.4 ^b,A^	34.6 ± 0.6 ^c,A^	24 ± 2 ^b,A^	123.989 ***
	2	37 ± 3 ^a,B^	49.2 ± 0.4 ^b,B^	68 ± 3 ^c,B^	44 ± 0.2 ^d,B^	124.295 ***
	4	68 ± 5 ^a,C^	97 ± 5 ^b,C^	122 ± 6 ^c,C^	98 ± 5 ^b,C^	53.616 ***
	F_3,6_	181.023 ***	432.873 ***	397.225 ***	504.720 ***	

Values are displayed as averages ± standard deviation (n = 3). For every soil treatment and for every IS ratio, one-way ANOVA was executed, and test outcomes (F) are presented in related lines as ***—significant at *p* < 0.001 level. Averages for equal soil treatment and IS ratio exhibiting diverse lowercase letters are significantly (*p* < 0.05) different from one another in accordance with the Duncan test. For every soil treatment and every plant tissue, one-way ANOVA was also executed, and test outcomes (F) are presented in related columns and as ***—significant at *p* < 0.001 level. Averages for the same soil treatment and plant tissue presenting diverse uppercase letters are significantly (*p* < 0.05) different from one another in accordance with the Duncan test.

**Table 6:** Biogas IPR (mL/day) (initial production rate) obtained in all BMP assays (average ± SD).

Treatment	IS Ratio	Root	Stem	Cob	Plant	F_4,8_
**Agricultural (A)**	1	7.3 ± 0.3 ^a,A^	10.6 ± 0.5 ^b,A^	9.0 ± 0.4 ^c,A^	10.6 ± 0.9 ^b,A^	25.749 ***
	2	9.2 ± 0.5 ^a,B^	13.1 ± 0.5 ^b,B^	12.5 ± 0.6 ^b,A^	12 ± 2 ^b,A^	7.957 ***
	4	12.3 ± 0.6 ^a,C^	19.8 ± 0.8 ^b,C^	31 ± 6 ^c,B^	22 ± 2 ^b,B^	17.975 ***
	F_3,6_	86.914 ***	165.298 ***	37.804 ***	31.206 ***	
**Industrial (I)**	1	7.8 ± 0.2 ^a,A^	10.5 ± 0.3 ^bc,A^	9.6 ± 0.2 ^b,A^	11 ± 1 ^c,A^	16.147 ***
	2	8.7 ± 0.2 ^a,A^	13.0 ± 0.2 ^b,B^	14.2 ± 0.3 ^c,B^	16.07 ± 0.06 ^d,B^	770.380 ***
	4	12 ± 2 ^a,B^	19 ± 1 ^b,C^	27 ± 4 ^c,C^	17.4 ± 0.1 ^b,C^	25.608 ***
	F_3,6_	13.265 **	86.920 ***	52.344 ***	76.350 ***	

Values are displayed as averages ± standard deviation (n = 3). For every soil treatment and for every IS ratio, test outcomes (F) are presented in the related lines as ***—significant at *p* < 0.001 level. Averages for equal soil treatment and IS ratio exhibiting diverse lowercase letters are significantly (*p* < 0.05) different from one another in accordance with the Duncan test. For every soil treatment and every plant tissue, one-way ANOVA was also executed, and test outcomes (F) are presented in related columns and as **—significant at *p* < 0.01 level and ***—significant at *p* < 0.001 level, correspondingly. Averages for the same soil treatment and plant tissue presenting diverse uppercase letters are significantly (*p* < 0.05) different from one another in accordance with the Duncan test.

**Table 7:** Methane volume (mL/batch) obtained at the end of BMP assays (average ± SD).

Treatment	IS Ratio	Root	Stem	Cob	Plant	F_4,8_
**Agricultural (A)**	1	10.6 ± 0.6 ^a,A^	16.2 ± 0.7 ^b,A^	21.7 ± 0.8 ^c,A^	16.5 ± 0.2 ^b,A^	171.633 ***
	2	19 ± 2 ^a,B^	35 ± 1 ^b,B^	34 ± 2 ^b,B^	38 ± 3 ^c,B^	59.834 ***
	4	43 ± 2 ^a,C^	65.9 ± 0.3 ^b,C^	78 ± 5 ^c,C^	65 ± 2 ^b,C^	87.241 ***
	F_3,6_	512.711 ***	2314.689 ***	306.878 ***	422.449 ***	
**Industrial (I)**	1	11.3 ± 0.3 ^a,A^	17.0 ± 0.7 ^b,A^	18 ± 1 ^b,A^	18.1 ± 0.9 ^b,A^	42.077 ***
	2	21.0 ± 0.2 ^a,B^	31.1 ± 0.5 ^b,B^	42 ± 2 ^c,B^	32.0 ± 0.7 ^d,B^	205.525 ***
	4	45 ± 7 ^a,C^	60 ± 2 ^b,C^	76 ± 3 ^c,C^	63 ± 1 ^b,C^	29.103 ***
	F_3,6_	54.076 ***	1402.332 ***	503.713 ***	1799.759 ***	

Values are displayed as averages ± standard deviation (n = 3). For every soil treatment and for every IS ratio, one-way ANOVA was executed, and test outcomes (F) are presented in related lines as ***—significant at *p* < 0.001 level. Averages for equal soil treatment and IS ratio exhibiting diverse lowercase letters are significantly (*p* < 0.05) different from one another in accordance with the Duncan test. For every soil treatment and every plant tissue, one-way ANOVA was also executed, and test outcomes (F) are presented in related columns and as ***—significant at *p* < 0.001 level. Averages for the same soil treatment and plant tissue presenting diverse uppercase letters are significantly (*p* < 0.05) different from one another in accordance with the Duncan test.

**Table 8:** Methane percentage in the biogas obtained at the end of BMP assays (average ± SD).

Treatment	IS Ratio	Root	Stem	Cob	Plant	F_4,8_
**Agricultural (A)**	1	70 ± 8 ^a,A^	64 ± 4 ^a,A^	63.7 ± 0.9 ^a,A^	70 ± 2 ^a,A^	1.984 NS
	2	61 ± 2 ^a,A^	66 ± 1 ^b,A^	53.3 ± 0.7 ^c,B^	67 ± 3 ^b,A^	31.983 ***
	4	72 ± 7 ^a,A^	63 ± 2 ^b,A^	60 ± 2 ^b,C^	62.0 ± 0.8 ^b,B^	5.538 *
	F_3,6_	2.505 NS	0.769 NS	37.024 ***	10.965 **	
**Industrial (I)**	1	65 ± 3 ^a,B^	68 ± 3 ^a,B^	53 ± 3 ^b,A^	76 ± 6 ^c,A^	20.302 ***
	2	57 ± 4 ^a,A^	63.1 ± 0.9 ^b,A^	60.9 ± 0.3 ^b,B^	73 ± 2 ^c,A^	27.698 ***
	4	62 ± 1 ^a,B^	62 ± 3 ^a,A^	62.0 ± 0.9 ^a,B^	65 ± 3 ^a,B^	1.452 NS
	F_3,6_	6.309 *	5.365 *	23.970 ***	7.660 *	

Values are displayed as averages ± standard deviation (n = 3). For every soil treatment and for every IS ratio, one-way ANOVA was executed, and test outcomes (F) are presented in related lines as NS—non-significant at *p* < 0.05 level; *—significant at *p* < 0.05 level; and ***—significant at *p* < 0.001 level, correspondingly. Averages for equal soil treatment and IS ratio exhibiting diverse lowercase letters are significantly (*p* < 0.05) different from one another in accordance with the Duncan test. For every soil treatment and every plant tissue, one-way ANOVA was also executed, and test outcomes (F) are presented in related columns and as NS—non-significant at *p* < 0.05 level. *—significant at *p* < 0.05 level; **—significant at *p* < 0.01 level; and ***—significant at *p* < 0.001 level, correspondingly. Averages for the same soil treatment and plant tissue presenting diverse uppercase letters are significantly (*p* < 0.05) different from one another in accordance with the Duncan test.

**Table 9:** Biogas yield (mL Biogas g^−^^1^ VS) obtained at the end of BMP assays (average ± SD).

Treatment	IS Ratio	Root	Stem	Cob	Plant	F_4,8_
**Agricultural (A)**	1	170 ± 12 ^a,A^	281 ± 16 ^b,A^	383 ± 16 ^c,A^	263 ± 6 ^b,A^	133.665 ***
	2	173 ± 9 ^a,A^	297 ± 13 ^b,A^	357 ± 21 ^c,A^	317 ± 27 ^b,B^	52.258 ***
	4	169 ± 11 ^a,A^	292 ± 7 ^b,A^	364 ± 17 ^c,A^	294 ± 5 ^b,AB^	155.465 ***
	F_3,6_	0.119 NS	1.154 NS	1.511 NS	8.439 *	
**Industrial (I)**	1	197 ± 12 ^a,A^	280 ± 4 ^b,A^	389 ± 7 ^c,A^	267 ± 20 ^b,A^	123.989 ***
	2	210 ± 16 ^a,A^	277 ± 2 ^b,A^	387 ± 17 ^c,A^	246.9 ± 0.6 ^d,A^	124.295 ***
	4	192 ± 13 ^a,A^	272 ± 15 ^b,A^	343 ± 17 ^c,B^	276 ± 14 ^b,A^	53.616 ***
	F_3,6_	1.293 NS	0.642 NS	10.106 *	3.315 NS	

Values are displayed as averages ± standard deviation (n = 3). For every soil treatment and for every IS ratio, one-way ANOVA was executed, and test outcomes (F) are presented in related lines as ***—significant at *p* < 0.001 level. Averages for equal soil treatment and IS ratio exhibiting diverse lowercase letters are significantly (*p* < 0.05) different from one another in accordance with the Duncan test. For every soil treatment and every plant tissue, one-way ANOVA was also executed, and test outcomes (F) are presented in related columns and as NS—non-significant at *p* < 0.05 level and *—significant at *p* < 0.05 level, correspondingly. Averages for the same soil treatment and plant tissue presenting diverse uppercase letters are significantly (*p* < 0.05) different from one another in accordance with the Duncan test.

**Table 10:** Methane yield (mL CH_4_ g^−^^1^ VS) obtained at the end of BMP assays (average ± SD).

Treatment	IS Ratio	Root	Stem	Cob	Plant	F_4,8_
**Agricultural (A)**	1	119 ± 7 ^a,AB^	182 ± 8 ^b,A^	244 ± 8 ^c,A^	185 ± 2 ^b,A^	171.633 ***
	2	107 ± 7 ^a,B^	195 ± 8 ^b,A^	191 ± 10 ^b,B^	213 ± 17 ^c,B^	59.834 ***
	4	121 ± 5 ^a,A^	185.1 ± 0.7 ^b,A^	220 ± 13 ^c,C^	183 ± 5 ^b,A^	87.241 ***
	F_3,6_	4.610 NS	3.391 NS	19.704 **	7.917 *	
**Industrial (I)**	1	127 ± 20 ^a,A^	190 ± 7 ^b,B^	204 ± 15 ^b,A^	203 ± 10 ^b,A^	42.077 ***
	2	118 ± 1 ^a,A^	175 ± 3 ^b,A^	236 ± 11 ^c,B^	180 ± 4 ^b,B^	205.525 ***
	4	127 ± 20 ^a,A^	168 ± 4 ^b,A^	212 ± 9 ^c,A^	178 ± 3 ^b,B^	29.103 ***
	F_3,6_	0.596 NS	14.727 **	5.864 *	13.763 *	

Values are displayed as averages ± standard deviation (n = 3). For every soil treatment and for every IS ratio, one-way ANOVA was executed, and test outcomes (F) are presented in related lines as ***—significant at *p* < 0.001 level. Averages for equal soil treatment and IS ratio exhibiting diverse lowercase letters are significantly (*p* < 0.05) different from one another in accordance with the Duncan test. For every soil treatment and every plant tissue, one-way ANOVA was also executed, and test outcomes (F) are presented in related columns and as NS—non-significant at *p* < 0.05 level; *—significant at *p* < 0.05 level; and **—significant at *p* < 0.01 level, correspondingly. Averages for the same soil treatment and plant tissue presenting diverse uppercase letters are significantly (*p* < 0.05) different from one another in accordance with the Duncan test.

**Table 11:** Soil properties (analytical method) [29].

Parameter	Agricultural	Industrial	(Methodology)
pH (1:2.5)	6.52 ± 0.08	5.80 ± 0.06	(potentiometry)
Organic matter (%)	3.0 ± 0.3	7.2 ± 0.1	(Walkley–Black method)
Total nitrogen (mg kg^−1^)	1248 ± 62	2602 ± 968	(Kjeldahl method)
Total phosphorous (mg kg^−1^)	1628 ± 34	2400 ± 23	(colorimetric–ascorbic acid method)
Extractable potassium (mg kg^−1^)	98 ± 14	41 ± 12	(Egner–Rhein method)
Extractable magnesium (mg kg^−1^)	101 ± 11	45 ± 11	(ammonium acetate)
Total zinc (mg kg^−1^)	37 ± 3	599 ± 12	(aqua regia–FAAS)
Total cadmium (mg kg^−1^)	0.5 ± 0.5	1.2 ± 0.5	(aqua regia–FAAS)

## Data Availability

The data presented in this study are available in the article.
